# Public mental health: Required actions to support implementation

**DOI:** 10.1192/j.eurpsy.2025.188

**Published:** 2025-08-26

**Authors:** J. Campion

**Affiliations:** SOUTH LONDON AND MAUDSLEY NHS FOUNDATION TRUST, London, United Kingdom

## Abstract

**Abstract:**

Jonathan will begin by outlining the impact of mental disorder and wellbeing. He will summarise effective public mental health interventions to treat mental disorder, prevent associated impacts, prevent mental disorder, and promote mental wellbeing and resilience. He will outline the scale of implementation failure of such interventions and the reasons for this. He will then set out required actions and real-world solutions to support scale implementation of public mental health interventions leading to sustainable reduction of the impact of mental disorder and the promotion of mental wellbeing.

**Disclosure of Interest:**

None Declared

